# Subcutaneous Phaeohyphomycosis caused by the rare emerging fungus *Parathyridaria percutanea*

**DOI:** 10.22034/cmm.2026.345496.1731

**Published:** 2026-02-01

**Authors:** Manharpreet Kaur, Haseen Ahmad, Manpreet Kaur, Satwinderpal Singh, Harsimran Kaur, Rupinder Singh, Mani Bhushan Kumar, Shivaprakash M Rudramurthy

**Affiliations:** 1 Assistant Professor, Department of Microbiology, Christian Medical College and Hospital, Ludhiana, Punjab, India; 2 Specialist Medical Officer Department of Microbiology, Civil Hospital, Nawanshahr, Punjab, India; 3 Ph.D. Scholar, Department of Medical Microbiology, Postgraduate Institute of Medical Education and Research, Chandigarh, India; 4 Specialist Medical Officer, Department of Pathology, Civil Hospital, Nawanshahr, Punjab, India; 5 Senior Medical Officer, Department of General Surgery, Civil Hospital, Nawanshahr, Punjab, India; 6 Additional Professor, Department of Medical Microbiology, Postgraduate Institute of Medical Education and Research, Chandigarh, India; 7 Research Assistant, Department of Microbiology, Civil Hospital, Nawanshahr, Punjab, India; 8 Assistant Professor, Department of Microbiology, Christian Medical College and Hospital, Ludhiana, Punjab, India; 9 Professor, Department of Medical Microbiology, Postgraduate Institute of Medical Education and Research, Chandigarh, India

**Keywords:** *Dematiaceous* hyphae, Itraconazole, Swelling

## Abstract

**Background and Purpose:**

*Parathyridaria percutanea* is an emerging human fungal pathogen that primarily causes subcutaneous phaeohyphomycosis. Molecular sequencing is an accurate and definitive method for identification. Treatment can be achieved through either surgical intervention or antifungal therapy.

**Case Presentation:**

This study aimed to report a rare case of *Parathyridaria percutanea* subcutaneous phaeohyphomycosis in a 62-year-old diabetic woman presenting with a forearm swelling. Diagnosis was confirmed by molecular sequencing after culture yielded non-sporulating hyphae. The patient underwent surgical excision followed by short-course itraconazole, with complete resolution and no recurrence on one-year follow-up.

**Conclusion:**

This case highlighted that *P. percutanea* subcutaneous phaeohyphomycosis is a rare organism isolated from a clinical case. In cases where non-sporulating morphology delays diagnosis, molecular tools provide reliable identification, while surgical excision with itraconazole ensures effective management.

## Introduction

*Parathyridaria percutanea* is an opportunistic and emerging dematiaceous fungus that has become an emerging cause of subcutaneous phaeohyphomycosis in renal transplant recipients in India [1]. Formerly known as *Roussoella percutanea*, it belongs to the order *Pleosporales* [2]. Limited cases of subcutaneous infections caused by *P. percutanea* have been reported in immunosuppressed hosts, such as renal transplant recipients, patients with Cushing’s syndrome, interstitial lung disease, and diabetes. A rare case of mycotic endophthalmitis has also been reported in an immunocompetent patient [1,3]. The route of infection is typically through traumatic implantation, particularly in individuals who work in fields or walk barefoot. *Parathyridaria percutanea* is a rare cause of subcutaneous phaeohyphomycosis, with very few cases reported in immunocompetent hosts. This case highlighted its typical presentation as a subcutaneous swelling and its successful management with surgical excision and itraconazole in an immunocompetent woman.

## Case Presentation

This study aimed to describe a rare case of *Parathyridaria percutanea* subcutaneous phaeohyphomycosis in a 62-year-old female from Punjab. She presented with a localized swelling over the medial aspect of the distal right forearm, approximately 6 cm proximal to the wrist joint, for the past three months (Figure 1A). The swelling had gradually increased in size over this period. On examination, the swelling measured 18 × 16 mm and was non-tender, non-pulsatile, normothermic, and firm in consistency. It was fixed to the underlying subcutaneous fascia but was movable in the vertical direction. The patient had no history of animal bites and could not recall any injury caused by vegetative matter. She had a six-year history of diabetes and hypertension and was on oral hypoglycemic and antihypertensive medications, respectively. Her vital signs were within the normal range.

**Figure 1:**
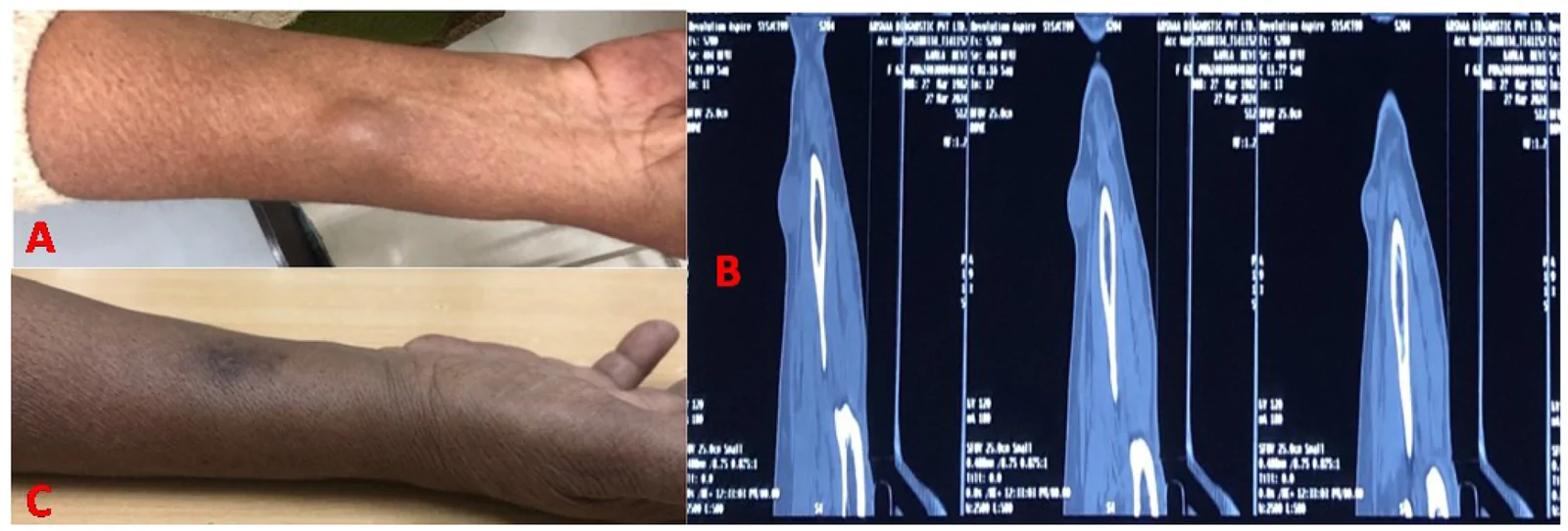
**A and C.** Pre-operative and post-operative clinical photograph of the patient. **B.** Non-contrast computed tomography shows a hypodense lesion over the subcutaneous plane of the distal right forearm.

A non-contrast computed tomography (NCCT) scan of the swelling was performed, which revealed a well-defined hypodense lesion in the subcutaneous plane, with no evidence of muscular extension (Figure 1B). The underlying bones appeared normal, with no signs of calcification, and no fat attenuation component was noted. Fine-needle aspiration (FNA) of the swelling was performed, and thick, straw-colored fluid was sent for cytological and microbiological examination.

Giemsa staining revealed necrotic debris with a few inflammatory cells, predominantly lymphocytes and some neutrophils, along with numerous septate hyphae (Figure 2A). 10% potassium hydroxide (KOH) mount showed dematiaceous septate hyphae with irregular hyphal swellings. Culture on Sabouraud dextrose agar (SDA) yielded flat, whiteish colonies with sparse aerial hyphae after one week of incubation at 25°C, which later turned to cottony grayish-black (Figure 2B). Lactophenol cotton blue (LPCB) mount revealed only non-sporulating, septate, pigmented hyphae. Bacterial and mycobacterial examinations were non-contributory. The fungal isolate was subjected to the slide culture technique on Potato Dextrose Agar (PDA) and incubated at 28°C for up to two weeks, which still showed only pigmented septate hyphae (Figure 2C).

**Figure 2:**
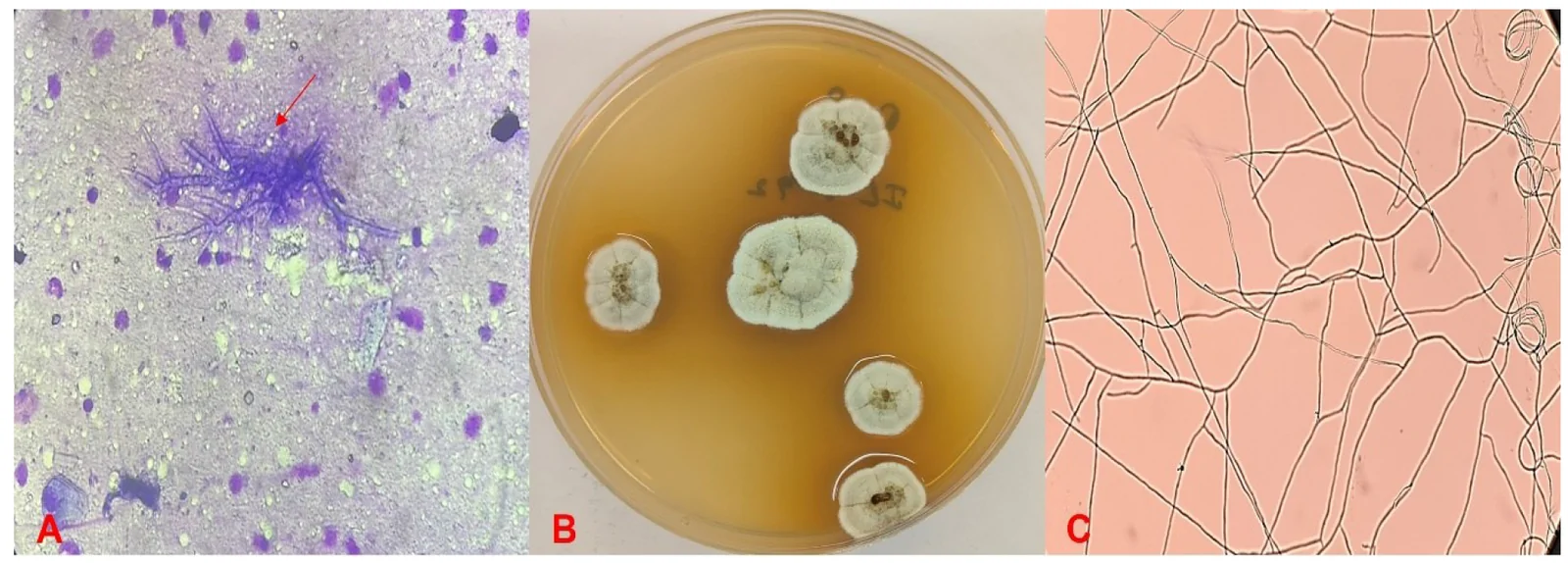
**A.** In Giemsa staining - necrotic debris with few inflammatory cells along with septate fungal hyphae, **B.** Cultural characteristics on Sabouraud dextrose agar, and **C.** Lactophenol cotton blue mount after performing slide culture.

Subsequently, the isolate was sent to the Post Graduate Institute of Medical Education and Research, Chandigarh, India, for molecular identification. Polymerase chain reaction (PCR) followed by Sanger sequencing of the isolate was performed using primers of the internal transcribed spacer (ITS4/5) region of rDNA. The analysis identified the isolate as *Parathyridaria percutanea*, showing 99.59% identity with the CBS 868.95 strain (GenBank accession no. NR_147631). The obtained isolate was submitted to the National Culture Collection of Pathogenic Fungi (NCCPF), Chandigarh, under the accession number NCCPF 104010, and the sequence was submitted to NCBI (GenBank accession no. PP833226).

The patient underwent wide excisional surgery for the swelling, followed by oral itraconazole at a dose of 200 mg twice daily for two months. However, she was compliant with the therapy for only 10 days. The patient was reviewed on day 3 for dressing change, on days 7 and 15 to assess wound healing, and subsequently at 30 days, 3 months, and then at 6-month intervals to monitor for recurrence of the swelling. She remained stable, and the entire lesion resolved on postoperative follow-up after 12 months (Figure 1C).

## Discussion

*Parathyridaria percutanea* is an emerging and opportunistic pathogen and a major causative agent of subcutaneous phaeohyphomycosis, particularly in renal transplant recipients [1]. It was first isolated in the year 2000 and misidentified as *Madurella mycetomatis*. However, accurate identification was achieved in 2014 when the organism was isolated from the swelling on the foot and ankle of a 45-year-old male and identified as *Roussoella percutanea* [4]. It remains a rare dematiaceous fungus reported from different parts of the world, with most of the cases originating from India and a few from the USA, UK, Africa, Europe, and Taiwan (Table 1).

**Table 1:** List of subcutaneous phaeohyphomycosis cases due to *Parathyridaria percutanea*.

Case no.	Author	Place	Year	Age/gender	Risk factor	Site of lesion	Treatment
1	Meis et al. [4]	The Netherlands	2000	63/M	Renal transplant (immunocompromised)	Foot	Surgical excision
2	Ahmed SA et al. [5]	USA	2014	45/M	Immunocompetent (Indian immigrant)	Leg	Surgical excision with Itraconazole
3	El Khalfi et al. [6]	France	2016	65/M	Renal-pancreatic transplant (immunocompromised)	Foot	Surgical excision with posaconazole
4	Almagro-Moltó M et al. [7]	Somalia	2017	55/F	Renal transplant (immunocompromised)	Knee	Surgical excision with Voriconazole
5	Vasant JA et al. [8]	UK	2017	47/M	Renal transplant (immunocompromised)	Leg	Surgical excision with Voriconazole
6	Rudramurthy SM et al. [1]	India (series)	2019	35-50/ M&F	Renal transplant (immunocompromised)	Hands and Feet	Surgical excision with Voriconazole
7	Yu et al. [9]	Taiwan	2020	81/M	Not available	Hand	Itraconazole (200 mg daily for 8 months)
8	Yadav B et al. [3]	India	2022	33/M	Immunocompetent	Eye	Voriconazole
9	Rafiq A et al. [10]	Somalia	2022	32/F	Renal transplant (immunocompromised)	Leg and hand	Surgical excision with itraconazole
10	Kaur H et al. [11]	India	2025	40/M	Renal transplant (immunocompromised)	foot	Surgical excision
11	Naharia P et al. [12]	India	2025	38/M	Immunocompromised	foot	Isavuconazole
12	Monpierre L et al. [13]	France	2025	63/F	Renal transplant (immunocompromised)	Foot	Isavuconazole
13	Keche A et al. [14]	India	2025	70/F	Immunocompetent	Hand	Itraconazole
14	Present study	India	2024	62/F	Immunocompetent	Hand	Surgical excision with itraconazole

There has been a noticeable increase in the number of reported subcutaneous phaeohyphomycosis cases caused by *P. percutanea*. To date, 17 cases have been documented in the literature—9 from India, 2 from Africa, and France, and 1 each from the UK, Taiwan, The Netherland, and the USA [1,3-14]. Infections typically occur in immunosuppressed patients; however, cases such as mycotic endophthalmitis reported by Yadav et al. and the subcutaneous phaeohyphomycosis reported by Keche et al. have also been described in immunocompetent individuals [3,14]. The majority of these cases involved patients with a history of renal transplantation, and rarely, Cushing’s syndrome, steroid abuse, or diabetes [1].

Traumatic inoculation is believed to be the primary route of infection. After inoculation, these fungi are typically dormant and multiply slowly in the subcutaneous tissue until the immune system of the host becomes compromised. A history of trauma was reported in only a few cases. In the present case, the patient had uncontrolled diabetes and was receiving oral hypoglycemic and antihypertensive medications. Presence of a painless swelling suggests that the pathogen may have remained dormant in the subcutaneous tissue before becoming clinically apparent. Identification of this fungus is phenotypically challenging due to its non-sporulating nature. Non-sporulating fungi can only be reliably identified through molecular sequencing, using pan-fungal primers targeting specific regions (internal transcribed spacer [ITS] or the large subunit (LSU)} of rDNA [1]. However, the high cost and limited availability of molecular methods often hinder diagnosis in resource-limited settings. In the present case, the isolate was identified by Sanger sequencing using ITS region primers of rDNA, as described previously [3]. According to joint guidelines from the European Society of Clinical Microbiology and Infectious Diseases (ESCMID), the Fungal Infection Study Group (EFISG), and the European Confederation of Medical Mycology (ECMM), subcutaneous phaeohyphomycosis should be treated with surgical excision followed by antifungal therapy [15]. Itraconazole and voriconazole are considered first-line therapies. Previous studies have shown that *P. percutanea* exhibits low minimum inhibitory concentrations to itraconazole, voriconazole, and posaconazole *in vitro* [5,7]. In the present case, the patient underwent surgical excision followed by itraconazole therapy. The swelling completely resolved, and no recurrence was observed during telephonic follow-up for up to 12 months. In the present case, antifungal susceptibility testing could not be performed due to the non-sporulating nature of the isolate.

Given the limited research available on the clinical spectrum and treatment of *P. percutanea* infections, this case may provide valuable insights and contribute meaningfully to the existing literature on comparable cases. Diagnosis of subcutaneous phaeohyphomycosis caused by *P. percutanea* in immunocompetent individuals is challenging. It requires microscopic examination, culture, and molecular techniques for complete diagnosis. Complete surgical excision combined with antifungal therapy is the mainstay of treatment.

## Conclusion

This case highlights the emergence of rarely implicated fungi, such as *P. percutanea*, in subcutaneous phaeohyphomycosis, particularly in diabetic patients without other immunocompromising conditions. Identification of such rare fungi is often delayed due to their non-sporulating nature, making molecular approaches the only reliable method for identification. However, the unavailability of molecular diagnostics in resource-limited settings contributes to the underdiagnosis of these infections. Surgical excision alone or in combination with itraconazole therapy remains the most effective treatment strategy.
